# Cooperative spreading processes in multiplex networks

**DOI:** 10.1063/1.4952964

**Published:** 2016-06-14

**Authors:** Xiang Wei, Shihua Chen, Xiaoqun Wu, Di Ning, Jun-an Lu

**Affiliations:** 1 School of Mathematics and Statistics, Wuhan University, Wuhan, Hubei 430072, China; 2 Department of Engineering, Honghe University, Honghe, Yunnan 661100, China; 3 Computational Science Hubei Key Laboratory, Wuhan University, Wuhan 430072, China; 4 School of Mathematics and Statistics, South-Central University for Nationalities, Wuhan 430074, China

## Abstract

This study is concerned with the dynamic behaviors of epidemic spreading in multiplex networks. A model composed of two interacting complex networks is proposed to describe cooperative spreading processes, wherein the virus spreading in one layer can penetrate into the other to promote the spreading process. The global epidemic threshold of the model is smaller than the epidemic thresholds of the corresponding isolated networks. Thus, global epidemic onset arises in the interacting networks even though an epidemic onset does not arise in each isolated network. Simulations verify the analysis results and indicate that cooperative spreading processes in multiplex networks enhance the final infection fraction.

In recent years, epidemic spreading has received considerable attention in multiplex networks. The main focus of studies on this process is on how epidemic spreads in multiplex networks when two spreading processes interact. Epidemic threshold and final infection fraction are the two important parameters underlying any spreading processes, to contrast the two parameters between the interacting networks, and the corresponding isolated networks has practical significance. In this study, a model for cooperative spreading processes on interacting two-layer networks is proposed. This study aims to prove that the epidemic thresholds of interacting two-layer networks can be decreased for cooperative spreading processes, which implies that cooperative spreading processes promote the spread of disease. This study determines that the global epidemic onset arises. Thus, a global epidemic threshold can be used to uniform the two epidemic thresholds. This model can also be used for unidirectional coupled networks. It is revealed that a network with a small epidemic threshold confirms the global epidemic threshold of the multiplex networks.

## I. INTRODUCTION

Epidemic spreading, which is an important dynamic process in complex networks, has attracted great attention for a long time. Many studies initially focused on isolated networks of fixed topology with mean-field approximation.^1,2^ The spread of sexually transmitted diseases and computer viruses was studied based on scale-free networks.^3–6^ Several studies also revealed that epidemic processes in scale-free networks do not pose an epidemic threshold because of large connectivity fluctuations in infinite scale-free networks.^7–9^ Epidemic spreading was investigated in complex random networks with degree correlations.^10^ Then, reaction-diffusion model in which the nodes contain agents^11–14^ was used in the process of spreading. The agents can move from a node to another, and spreading processes occurred within agents on the same node. Further, the studies focus on the mobility of individuals.^15,16^ The movement of individuals among dense crowd in a city has a great effect on the epidemic threshold. A random diffusion model was also used to investigate epidemic spreading among objective traveling people.^17,18^ The railway leads to a fast increase in the number of infected agents and a high final infection fraction. These models and results are very useful for public health authorities to make effective decisions.

However, many spreading processes have been studied independently in a single network. In the real-world, many spreading processes can occur through multiple routes simultaneously. For example, sexually transmitted diseases can spread both in homosexual and heterosexual networks.^19^ Avian influenza, such as 2009 H1N1 and 2013 H7N9, has recently made dead-end jumps from poultry to human.^20^ Thus, these viruses make challenges to human progress and survival. A natural extension is to use the interacting network model in which epidemic can spread from one network to another. Funk and Jansen^21^ used the bond percolation analysis of two competitive viruses in two-layer networks, and the effects of layer overlapping were investigated. A study on U.S. bisexual men showed that the bisexual men were the medium to connect the pathogens from the network of heterosexual men with the network of homosexual men.^22,23^ Dickison *et al*.^24^ studied the susceptible-infected-refractory (SIR) process and revealed that in strongly coupled networks, epidemics spread from one layer to the other at a critical infection strength *β_c_*, below which the disease does not spread. Saumell-Mendiola *et al*.^25^ analyzed an epidemic spreading process of two interacting networks. They also developed a heterogeneous mean-field approach and revealed that a global endemic state may arise in the coupled system even though two networks are well below their respective epidemic thresholds. Granell *et al*.^26^ used a microscopic Markov chain approach to analyze the interrelation between two processes, namely, the spreading of the epidemic and the spreading of information awareness to prevent infection. They also determined that awareness spread leads to disease suppression. Zhao *et al*.^27^ used bond percolation theory and the generating function with SIR model to calculate the epidemic thresholds of multiplex networks. Sahneh and Scoglio^28^ extended the single-virus spread model to the two exclusive viruses in two-layer networks, and they found analytical expressions that determine extinction, coexistence, and absolute dominance of the viruses. Zhao *et al*.^29^ promoted the concept of state-dependent infected rate, and all the different influences between the two epidemics were displayed. Therefore, epidemic spreading processes on top of multiplex networks reveal a rich phase diagram of intertwined effects.

Any spreading process has two underlying fundamental parameters: epidemic threshold and final infection fraction. The key issue is the difference in the two parameters between the interacting networks and the corresponding isolated networks. Cozzo *et al*.^30^ used perturbation theory to analyze epidemic thresholds of networks, and they revealed that the spectral radius of the whole matrix is always not less than that of each submatrix. Thus, the epidemic threshold of the whole system is always not more than the corresponding isolated networks. Wang *et al*.^31^ investigated two types of spreading dynamics: one is the spread of disease, whereas the other is the spread of information about the disease. They used simulation to determine that information spreading can effectively raise the epidemic threshold. Guo *et al*.^32^ proposed a local awareness controlled contagion spreading model in multiplex networks, and they determined the emergence of an abrupt transition of epidemic threshold with the local awareness ratio through numerical simulations.

The study on the interaction between two spread processes on two-layer networks has a great significance. In this study, a model composed of two interacting complex networks is proposed to describe the cooperative spreading processes. First, this model extends spreading process in a single network to cooperative spreading in the two-layer networks. It can also be used to analyze unidirectional or bidirectional interaction between two-layer networks. Second, perturbation analysis theory is used to prove that epidemic thresholds of interacting two-layer networks can be decreased in cooperative spreading processes. This theory reveals that the cooperative interaction between two spread processes can promote spread. Epidemic onset arises in one network and that also arises simultaneously in the other network for the interacting networks. Thus, a global epidemic threshold for interacting networks exists. Finally, this theory also displays that cooperative spreading processes in multiplex network enhance the final infection fraction.

The rest of this paper is organized as follows. A model composed of two interacting complex networks and theoretical analysis is proposed in Sec. II. Then, the numerical simulations are used to show the effects of the interactions between two spreading processes in Sec. III. Finally, some discussions and conclusions are given in Sec. IV.

## II. NETWORK MODELING AND PRELIMINARIES

**FIG. 1.**
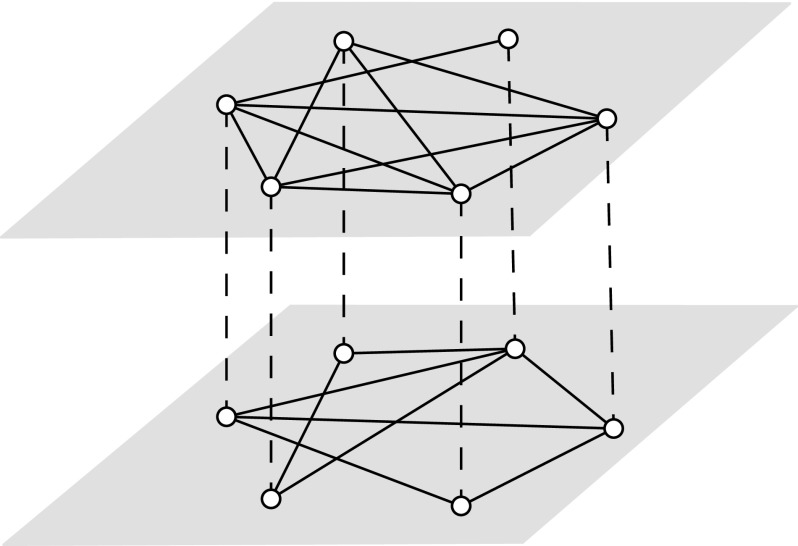
Example of a two-layer network. The topologies are different per layer, and each node in one layer is connected to its counterparts in the other layer. Dashed and solid lines represent interlayer and intralayer connections, respectively.

Interacting two-layer networks, including network $A={\left(a_{ij}\right)}_{N\times N}$ and $B={\left(b_{ij}\right)}_{N\times N}$, presumably have the same size *N* with different intra-layer connectivity. Thus, the model is a multiplex networks, as shown in Fig. 1. We focus in the discrete standard susceptible-infected-susceptible (SIS) model^1^ for both networks with regard to the spreading dynamics. In each subnetwork, the nodes are denoted as susceptible (S) or infected (I), and the links represent the connection along which the infection can propagate. At each time step, susceptible (S) nodes may be infected from intra-layer infected nodes and inter-layer infected nodes simultaneously. On the other hand, infected nodes recover spontaneously. After some transient time, the previous dynamics make the system into a stationary state. For interacting two-layer networks, let *β*_1_(*β*_2_) be the probability of infection between nodes in network *A*(*B*) and *γ*_1_(*γ*_2_), the probability of infection from a node in *B*(*A*) to a node in *A*(*B*), *μ*_1_(*μ*_2_) is the probability of curing for network *A*(*B*). The prevalence of infection of *A* and *B*, defined as the fraction of infected nodes at a given time, is denoted by $p_{1,i}(t)$ and $p_{2,i}(t)$ for node *i*, respectively. So, the time evolution processes can be written as

$$\begin{array}{c} p_{1,i}(t+1)=(1-p_{1,i}(t))(1-q_{1,i}(t))\\ +(1-\mu_{1})p_{1,i}(t)+\gamma_{1}p_{2,i}(t)(1-p_{1,i}(t)),\\ p_{2,i}(t+1)=(1-p_{2,i}(t))(1-q_{2,i}(t))\\ +(1-\mu_{2})p_{2,i}(t)+\gamma_{2}p_{1,i}(t)(1-p_{2,i}(t)), \end{array}$$ (1)

for i∈{1,...,N}, where $q_{1,i}(t)$ and $q_{2,i}(t)$ are the probabilities of node *i* not being infected by any neighbor in *A* and *B*, respectively

$$\begin{array}{c} q_{1,i}(t)=\prod_{j=1}^{N}\left(1-\beta_{1}a_{ij}p_{1,j}(t)\right),\\ q_{2,i}(t)=\prod_{j=1}^{N}\left(1-\beta_{2}b_{ij}p_{2,j}(t)\right). \end{array}$$ (2)

In the first line in Eq. (1), the first term on the right-hand side is the probability that node *i* is susceptible $\left(1-p_{1,i}(t)\right)$ and is infected $\left(1-q_{1,i}(t)\right)$ by at least a neighbor, the second term stands for the probability that node *i* is infected at time *t* and does not recover, and the last term takes into account the probability that node *i* is susceptible $\left(1-p_{1,i}(t)\right)$ and is infected by the counterpart node in other layer, the second line in Eq. (1) has the same definition with the first line. Based on different epidemic parameters, this model can construct various network structures to exhibit rich propagation dynamic behaviors.

In the following, a simplifying assumption should be made. The probability of infection of inter-layer is much smaller than the probability of infection of intra-layer,^30^ since the probability of infection of inter-layer describes spreading process from one species to another species.^20^ So, one obtains:

$$\begin{array}{c} \gamma_{1}\ll \beta_{1},\\ \gamma_{2}\ll \beta_{2}. \end{array}$$ (3)

The fixed point iteration method is used to calculate nontrivial stationary solution of Eq. (1)

$$\begin{array}{c} p_{1,i}=(1-p_{1,i})(1-q_{1,i})+(1-\mu_{1})p_{1,i}\\ +\gamma_{1}p_{2,i}(1-p_{1,i}),\\ p_{2,i}=(1-p_{2,i})(1-q_{2,i})+(1-\mu_{2})p_{2,i}\\ +\gamma_{2}p_{1,i}(1-p_{2,i}), \end{array}$$ (4)

where

$$\begin{array}{c} q_{1,i}=\prod_{j=1}^{N}\left(1-\beta_{1}a_{ij}p_{1,j}\right)\\ q_{2,i}=\prod_{j=1}^{N}\left(1-\beta_{2}b_{ij}p_{2,j}\right). \end{array}$$ (5)

$p_{1,i}$ and $p_{2,i}$ are the probability of infection of node *i* at the stationary state for *A* and *B*, respectively. Thus, the final infection fractions *ρ*_1_ and *ρ*_2_ for the two interacting networks *A* and *B* are computed as

$$\rho_{i}=\frac{1}{N}\sum_{j=1}^{N}p_{i,j},\;i=1,2.$$ (6)

When the values of *μ_i_* and *γ_i_* are fixed, there are epidemic thresholds $\beta_{1}^{c}$ and $\beta_{2}^{c}$ for *A* and *B*, respectively, thus $\rho_{1}=0$ if $\beta_{1}<\beta_{1}^{c}$, and $\rho_{2}=0$ if $\beta_{2}<\beta_{2}^{c}$. $\rho_{1}>0$ if $\beta_{1}>\beta_{1}^{c}$, and $\rho_{2}>0$ if $\beta_{2}>\beta_{2}^{c}$. When $\beta_{1}\to \beta_{1}^{c}$ and $\beta_{2}\to \beta_{2}^{c}$, the probabilities $p_{1,i}\ll 1$ and $p_{2,i}\ll 1$, so from Eq. (2), one obtains

$$\begin{array}{c} q_{1,i}\approx 1-\beta_{1}\sum_{j=1}^{N}a_{ij}p_{1,j},\\ q_{2,i}\approx 1-\beta_{2}\sum_{j=1}^{N}b_{ij}p_{2,j}. \end{array}$$ (7)

Inserting Eq. (7) into Eq. (4) and neglecting second-order terms, one obtains

$$\begin{array}{c} \beta_{1}\sum_{j=1}^{N}a_{ij}p_{1,j}-\mu_{1}p_{1,i}+\gamma_{1}p_{2,i}=0,\\ \beta_{2}\sum_{j=1}^{N}b_{ij}p_{2,j}-\mu_{2}p_{2,i}+\gamma_{2}p_{1,i}=0, \end{array}$$ (8)

thus, we obtain the following equation:

$$\begin{array}{c} \beta_{1}AP_{1}-\mu_{1}P_{1}+\gamma_{1}P_{2}=0,\\ \beta_{2}BP_{2}-\mu_{2}P_{2}+\gamma_{2}P_{1}=0, \end{array}$$ (9)

where $P_{1}={\left[p_{1,1},p_{1,2},...,p_{1,N}\right]}^{⊤},P_{2}={\left[p_{2,1},p_{2,2},...,p_{2,N}\right]}^{⊤}$. From the second line of Eq. (9), we obtain

$$(B-\frac{\mu_{2}}{\beta_{2}}I)P_{2}=-\frac{\gamma_{2}}{\beta_{2}}P_{1},$$ (10)

when $\frac{\mu_{2}}{\beta_{2}}$ is not the eigenvalue of matrix B, then matrix ($B-\frac{\mu_{2}}{\beta_{2}}I$) is reversible, note that irreversible measure of ($B-\frac{\mu_{2}}{\beta_{2}}I$) is zero, since the number of eigenvalues for matrix *B* is limited. One obtains

$$P_{2}=-\frac{\gamma_{2}}{\beta_{2}}{\left(B-\frac{\mu_{2}}{\beta_{2}}I\right)}^{-1}P_{1}.$$ (11)

By the same methods, one obtains

$$P_{1}=-\frac{\gamma_{1}}{\beta_{1}}{\left(A-\frac{\mu_{1}}{\beta_{1}}I\right)}^{-1}P_{2}.$$ (12)

Inserting Eqs. (11) and (12) into Eq. (9), we obtain

$$\begin{array}{c} (A-\frac{\gamma_{1}\gamma_{2}}{\beta_{1}\beta_{2}}{\left(B-\frac{\mu_{2}}{\beta_{2}}I\right)}^{-1}-\frac{\mu_{1}}{\beta_{1}}I)P_{1}=0,\\ (B-\frac{\gamma_{1}\gamma_{2}}{\beta_{1}\beta_{2}}{\left(A-\frac{\mu_{1}}{\beta_{1}}I\right)}^{-1}-\frac{\mu_{2}}{\beta_{2}}I)P_{2}=0, \end{array}$$ (13)

after symbol substitution in Eq. (13), one gets

$$\begin{array}{c} (\bar{A}-\frac{\mu_{1}}{\beta_{1}}I)P_{1}=0,\\ (\bar{B}-\frac{\mu_{2}}{\beta_{2}}I)P_{2}=0, \end{array}$$ (14)

where

$$\begin{array}{c} \bar{A}=A-\frac{\gamma_{1}\gamma_{2}}{\beta_{1}\beta_{2}}{\left(B-\frac{\mu_{2}}{\beta_{2}}I\right)}^{-1},\\ \bar{B}=B-\frac{\gamma_{1}\gamma_{2}}{\beta_{1}\beta_{2}}{\left(A-\frac{\mu_{1}}{\beta_{1}}I\right)}^{-1}. \end{array}$$ (15)

When Eq. (14) has nonzero solution ($P_{1}>0,\;P_{2}>0$), if and only if $\mu_{1}/\beta_{1}$ is the eigenvalue of matrix $\bar{A}$ and $\mu_{2}/\beta_{2}$ is the eigenvalue of matrix $\bar{B}$.^10,33^ Looking for the onset of spread, the lowest values of *β*_1_ and *β*_2_ satisfying Eq. (14) are written as

$$\begin{array}{c} \beta_{1}^{c}=\frac{\mu_{1}}{\Lambda_{max}(\bar{A})},\\ \beta_{2}^{c}=\frac{\mu_{2}}{\Lambda_{max}(\bar{B})}, \end{array}$$ (16)

where Λ_*max*_ is the largest eigenvalue of the matrix.

Considering the two isolated networks *A* and *B*, probabilities of infection are denoted by $p_{1,i}(t)$ and $p_{2,i}(t)$ for node *i*, respectively

$$\begin{array}{c} p_{1,i}(t+1)=(1-p_{1,i}(t))(1-q_{1,i}(t))+(1-\mu_{1})p_{1,i}(t),\\ p_{2,i}(t+1)=(1-p_{2,i}(t))(1-q_{2,i}(t))+(1-\mu_{1})p_{2,i}(t). \end{array}$$ (17)

Inserting Eq. (2) into Eq. (17) and neglecting second-order terms, we can calculate nontrivial stationary solution for isolated networks by fixed point iteration method, one obtains

$$\begin{array}{c} (A-\frac{\mu_{1}}{\beta_{1}}I)P_{1}^{*}=0,\\ (B-\frac{\mu_{2}}{\beta_{2}}I)P_{2}^{*}=0. \end{array}$$ (18)

$P_{1}^{*}$ and $P_{2}^{*}$ are the probability of infection vectors for all nodes at the stationary state for *A* and *B*, respectively. Looking for the onset of spread ($P_{1}^{*}>0,\;P_{2}^{*}>0$), the lowest values of infected rate *β*_1_ and *β*_2_ for isolated networks satisfying Eq. (18) are

$$\begin{array}{c} \beta_{1}^{*}=\frac{\mu_{1}}{\Lambda_{max}(A)}\\ \beta_{2}^{*}=\frac{\mu_{2}}{\Lambda_{max}(B)}. \end{array}$$ (19)

Generally, probability of infection between layers is smaller than the probability of infection in the layer. Following the assumptions in Eq. (3), we obtain $\gamma_{1}\gamma_{2}\ll \beta_{1}\beta_{2}$. By comparing Eq. (13) with Eq. (18), we can set matrix $\frac{\gamma_{1}\gamma_{2}}{\beta_{1}\beta_{2}}{\left(B-\frac{\mu_{2}}{\beta_{2}}I\right)}^{-1}$ as a disturbance of the matrix *A*, and $\frac{\gamma_{1}\gamma_{2}}{\beta_{1}\beta_{2}}{\left(A-\frac{\mu_{1}}{\beta_{1}}I\right)}^{-1}$ as a disturbance of the matrix *B*. Therefore, in order to contrast the epidemic thresholds $\beta_{1}^{c}$ and $\beta_{2}^{c}$ of interacting networks with $\beta_{1}^{*}$ and $\beta_{2}^{*}$ of the corresponding isolated networks, we can use perturbation analysis method to analyze the thresholds of isolated networks. The perturbed solutions to both infected thresholds $\beta_{1}^{*}$ and $\beta_{2}^{*}$ and infection rates $P_{1}^{*}$ and $P_{2}^{*}$ of isolated networks are proposed as

$$\begin{array}{c} \beta_{1}^{c}=\beta_{1}^{*}+\epsilon_{1}\beta_{1}^{*}+O(\epsilon_{1}^{2})\\ \beta_{2}^{c}=\beta_{2}^{*}+\epsilon_{2}\beta_{2}^{*}+O(\epsilon_{2}^{2})\\ P_{1}=P_{1}^{*}+\epsilon_{3}P_{1}^{*}+O(\epsilon_{3}^{2})\\ P_{2}=P_{2}^{*}+\epsilon_{4}P_{2}^{*}+O(\epsilon_{4}^{2}). \end{array}$$ (20)

Inserting Eq. (20) into Eq. (9), using Eq. (19) and neglecting second-order terms, Eq. (9) after some algebra yields

$$\begin{array}{c} \epsilon_{1}\beta_{1}^{*}AP_{1}^{*}+\gamma_{1}(1+\epsilon_{4})P_{2}^{*}=0,\\ \epsilon_{2}\beta_{2}^{*}BP_{2}^{*}+\gamma_{2}(1+\epsilon_{3})P_{1}^{*}=0, \end{array}$$ (21)

since $|\epsilon_{3}|\ll 1$ and $|\epsilon_{4}|\ll 1$, *A* and *B* are adjacency matrixes, the results show that $\epsilon_{1}<0$ and $\epsilon_{2}<0$, one obtains $\beta_{1}^{c}<\beta_{1}^{*}$ and $\beta_{2}^{c}<\beta_{2}^{*}$. Therefore, we can conclude that the epidemic threshold of the interacting two-layer networks can be decreased for two cooperative spreading processes, which means that cooperative spreading promotes the spreading processes. To obtain more accurate results, we consider the second order approximation of Eq. (5) as follows:

$$\begin{array}{c} q_{1,i}\approx 1-\beta_{1}\sum_{j=1}^{N}a_{ij}p_{1,j}+\beta_{1}^{2}\sum_{j<l}a_{ij}a_{il}p_{1,j}p_{1,l},\\ q_{2,i}\approx 1-\beta_{2}\sum_{j=1}^{N}b_{ij}p_{2,j}+\beta_{2}^{2}\sum_{j<l}b_{ij}b_{il}p_{2,j}p_{2,l}. \end{array}$$ (22)

The second-order corresponds to reinfections and multiple infections.

The conclusion can be extended from duplex networks to multiplex networks. Take a multiplex network composed of three layers, for example. First, we consider a network which contains two layers of sub-networks. Based on the conclusion, we obtain that the epidemic threshold of this duplex network is decreased compared with the two corresponding isolated networks. Second, we take the duplex network as the first layer, and a third sub-network as the second layer. Similarly, based on the conclusion, we can conclude that the epidemic threshold of the multiplex network composed of three layers is decreased compared with the three corresponding isolated networks. Analogously, the conclusion can be generalized to multiplex networks composed of more layers of sub-networks.

It is observed that a global endemic activity arises in the two interacting complex networks, the reason being that the state $\rho_{1}\neq 0$ and $\rho_{2}=0$ is not a fixed point of the dynamics shown in Eq. (9). This conclusion is proved as below. The state $\rho_{1}\neq 0$ and $\rho_{2}=0$ is presumably a fixed point. Thus, the probability is $P_{1}>0$, and $P_{2}=0$. When $P_{2}=0$, one obtains $P_{1}=0$ after substitution into Eq. (9), wherein contradiction occurs. The state $\rho_{1}=0$ and $\rho_{2}\neq 0$ is also not a fixed point, as can be revealed with the same method. Thus, the fixed point is at the state $\rho_{1}\neq 0$ and $\rho_{2}\neq 0$. Thus, when $\beta_{2}\to \beta_{2}^{c}$, then $\beta_{1}\to \beta_{1}^{c}$ simultaneously. This finding indicates that if epidemic activity arises in one network, it will also arise in the other coupled network. That is to say, if the epidemic spreads in one of the coupled networks, it will spread to the whole system.

Generalized outer synchronization in unidirectional interaction of two networks has attracted great attention for a long time.^34^ However, the unidirectional interaction of two networks for an epidemic has not been considered thus far. The proposed model can also be used to analyze the unidirectional interaction of two networks by setting $\gamma_{1}=0$ or $\gamma_{2}=0$. In the unidirectional interaction of two-layer networks with $\gamma_{1}=0$, network *A* affects network *B*, but the inverse is not true. When the epidemic threshold of isolated network *A* is smaller than that of isolated network *B*, we call network *A* as the dominant network and network *B* as the nondominant network. The epidemic threshold of nondominant network *B* is proved smaller than that of corresponding isolated network with perturbation theory. Based on Eq. (9) with $\gamma_{1}=0$, the following can be obtained:

$$\begin{array}{c} \beta_{1}AP_{1}-\mu_{1}P_{1}=0,\\ \beta_{2}BP_{2}-\mu_{2}P_{2}+\gamma_{2}P_{1}=0. \end{array}$$ (23)

The perturbed solution is proposed to the infected threshold $\beta_{2}^{*}$ and the infection rate $P_{2}^{*}$ of the isolated network *B*

$$\begin{array}{c} \beta_{2}^{c}=\beta_{2}^{*}+\epsilon_{2}\beta_{2}^{*}+O(\epsilon_{2}^{2}),\\ P_{2}=P_{2}^{*}+\epsilon_{4}P_{2}^{*}+O(\epsilon_{4}^{2}). \end{array}$$ (24)

The following is obtained by inserting Eq. (24) into Eq. (23), the second line in Eq. (19), and by neglecting the second-order terms:

$$\epsilon_{2}\beta_{2}^{*}BP_{2}^{*}+\gamma_{2}P_{1}^{*}=0.$$ (25)

Given that *B* is adjacency matrix, the results show that $\epsilon_{2}<0$. Then, the following is obtained:

$$\beta_{2}^{c}<\beta_{2}^{*}.$$ (26)

This finding reveals that the epidemic threshold of *B* is well below that of the corresponding isolated network. The dominant network *A* with a small epidemic threshold confirms the global epidemic threshold of the multiplex networks, given that it induces a shift of the epidemic threshold of the network *B* to small values. In other words, the multiplex nature of the system leads to an earlier endemic activity also in the nondominant network, given that its epidemic threshold is smaller than that for the isolated networks.

## III. NUMERICAL SIMULATIONS

**FIG. 2.**
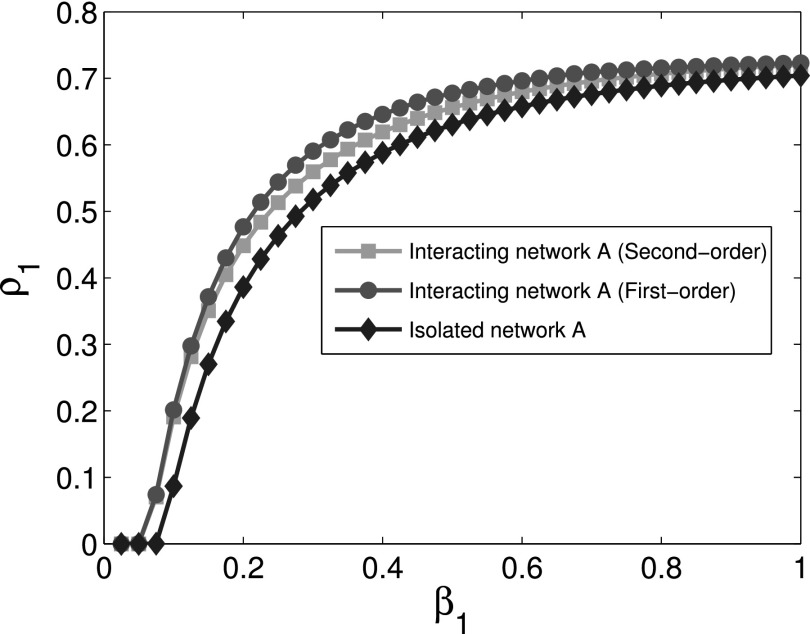
Comparison of the final infection fraction *ρ*_1_ as a function of *β*_1_ for cooperative network *A* with the corresponding isolated network.

**FIG. 3.**
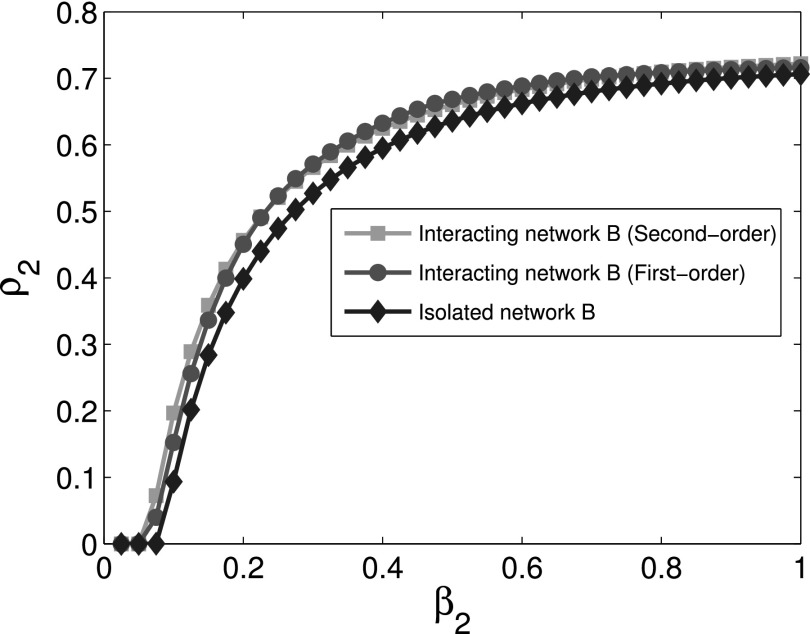
Comparison of the final infection fraction *ρ*_2_ as a function of *β*_2_ for cooperative network *B* with the corresponding isolated network.

**FIG. 4.**
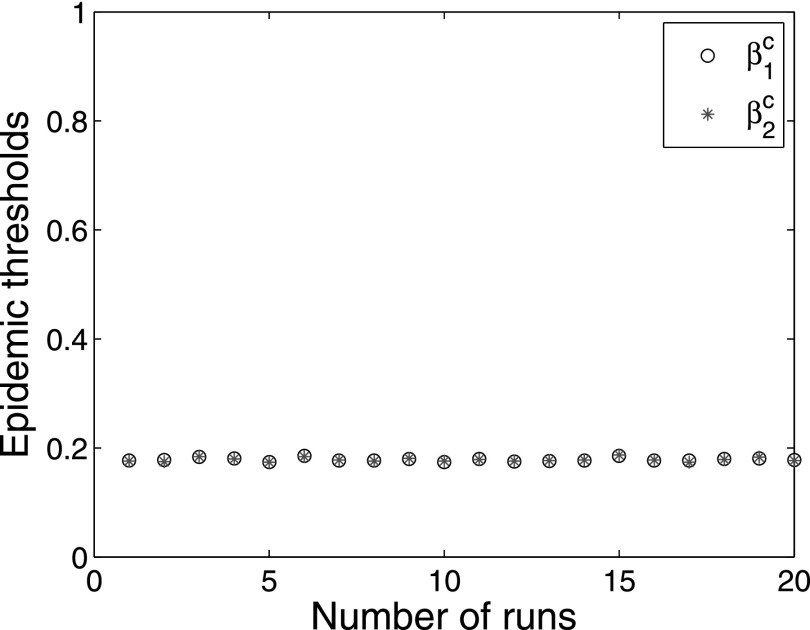
Comparison of the epidemic thresholds for two-layer interacting networks *A* and *B*. The x-axis represents the number of runs, and the y-axis represents the epidemic thresholds obtained by simulations for the two-layer interacting networks.

**FIG. 5.**
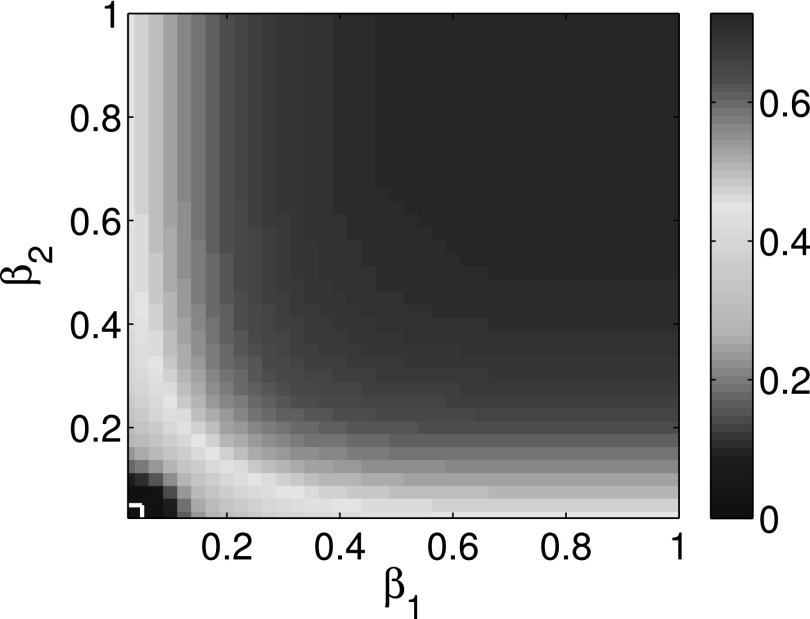
Expected infection ratios $\rho =(\rho_{1}+\rho_{2})/2$ for two-layer interacting networks as a function of probability of infection *β*_1_ and *β*_2_ using first order approximation.

**FIG. 6.**
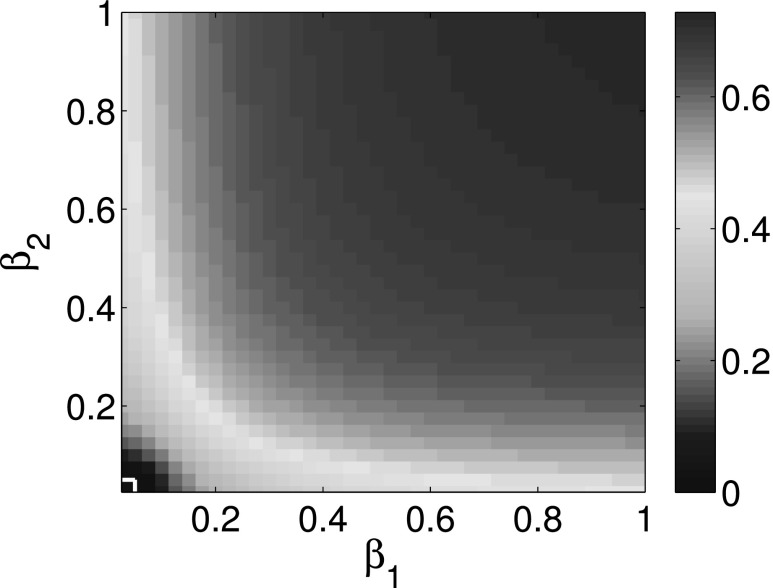
Expected infection ratios $\rho =(\rho_{1}+\rho_{2})/2$ for two-layer interacting networks as a function of probability of infection *β*_1_ and *β*_2_ by using second order approximation.

**FIG. 7.**
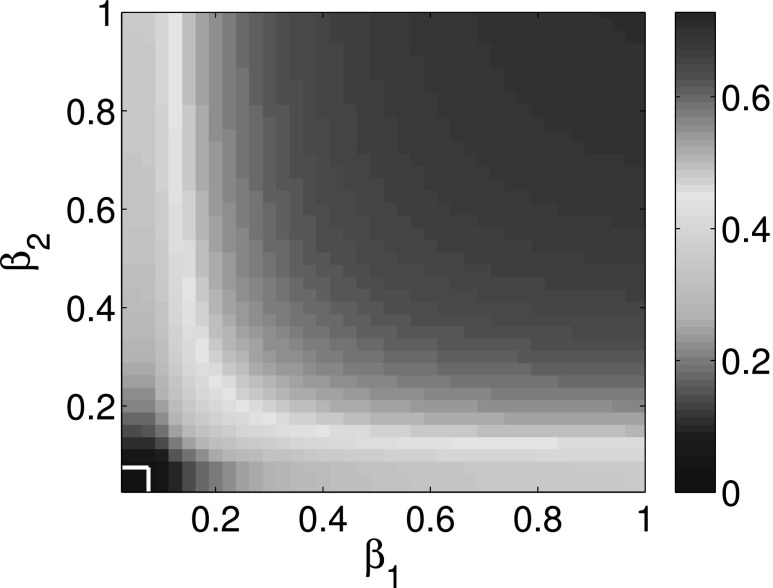
Expected infection ratios $\rho =(\rho_{1}+\rho_{2})/2$ for corresponding two isolated networks as a function of probability of infection *β*_1_ and *β*_2_.

**FIG. 8.**
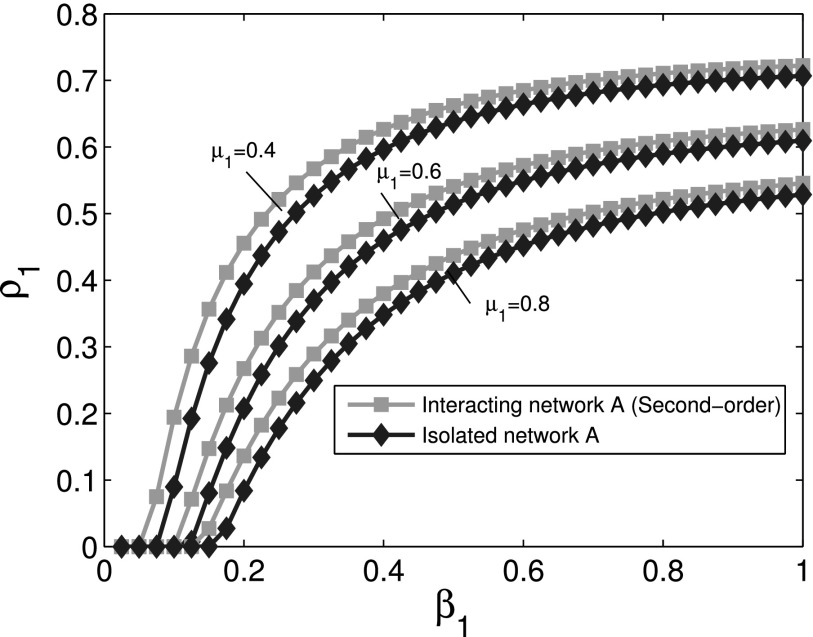
Comparison of the final infection fraction *ρ*_1_ as a function of *β*_1_ for cooperative network *A* with the corresponding isolated network for different values of *μ*_1_.

**FIG. 9.**
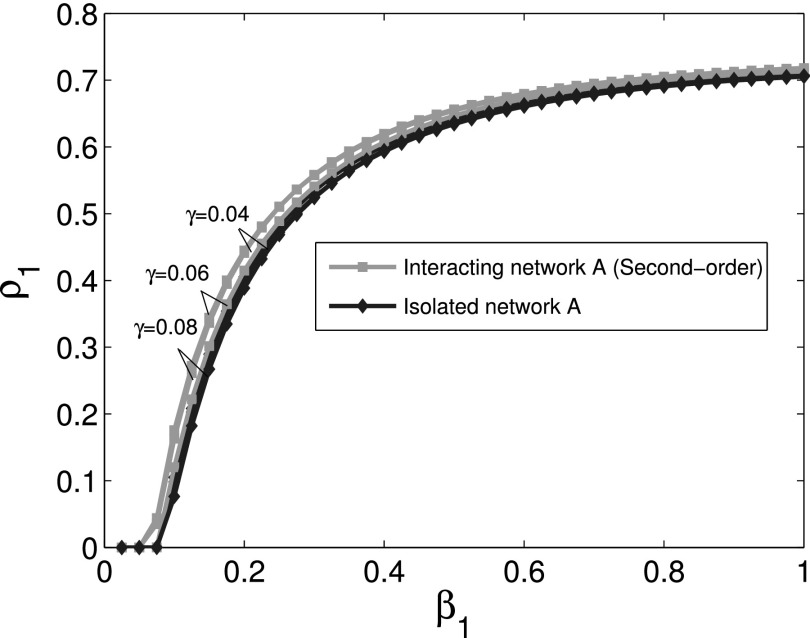
Comparison of the final infection fraction *ρ*_1_ as a function of *β*_1_ for cooperative network *A* with the corresponding isolated network for different values of *γ*.

In numerical simulations, two different networks formed by 1000 nodes each are generated, which follow the power law degree distribution with exponent 2.5, given that the epidemic thresholds are found at the limit for scale-free networks. Figs. 2–9 show results averaged over 20 runs of randomly generated two-layer models.

Monte Carlo simulations are employed to obtain the epidemic thresholds. The initial fraction of the infected nodes is set as 0.05, and the probability of curing are $\mu_{1}=0.4$ and $\mu_{2}=0.4$. The values for interlayer probability of infection are $\gamma_{1}=0.05$ and $\gamma_{2}=0.05$. For simplicity, we always set $\mu_{1}=\mu_{2}$ and $\gamma_{1}=\gamma_{2}$. Note that two additional types of topologies, two interacting random networks, and two interacting small-world networks are also used for simulations. The numerical results which are not shown here for brevity agree with those of the two interacting scale-free networks.

The comparison of the final infection fractions *ρ*_1_ and *ρ*_2_ of infected nodes for cooperative interaction networks with the corresponding isolated networks is shown in Figs. 2 and 3. Using first order approximation and second order approximation for cooperative spreading processes in numerical simulations, Figs. 2 and 3 show that the difference between first order approximation and second order approximation is trivial. It is also obvious that the epidemic thresholds are decreased for cooperative spreading processes compared with those of the corresponding isolated networks. This observation indicates that cooperative interacting networks promote propagation process. Figs. 2 and 3 also show that the final infection fraction for cooperative interaction networks is larger than that for isolated networks whether we use the first or second order approximation.

Further, the endemic activity of the two interacting complex networks arises simultaneously. Thus, the epidemic thresholds of the two interacting complex networks are always the same. Fig. 4 shows the comparison of epidemic thresholds for interacting networks *A* and *B*, where the x-axis represents the number of runs. In particular, red stars represent epidemic threshold $\beta_{1}^{c}$ for network *A*, and black circles represent epidemic threshold $\beta_{2}^{c}$ for network *B*. Fig. 4 shows that the epidemic thresholds $\beta_{1}^{c}$ are consistent with epidemic thresholds $\beta_{2}^{c}$ for 20 runs.

We call the sub-network with the smallest epidemic threshold as the dominant network. Numerical analysis is used to verify the conclusion indicating that spreading process in the dominant network can promote the spreading process in a nondominant network with unidirectional interaction. We assume that network *A* is dominant and network *B* is nondominant, and *A* affects *B* although the inverse is not the case. The simulation result which is not shown here for brevity is similar to Fig. 3. Thus, it reveals that the epidemic threshold of *B* in cooperative unidirectional interaction networks is well below the isolated network *B*. Therefore, we obtain the result with Eq. (26).

We provide three colormaps to illustrate the overall behavior. Figs. 5–7 show that the expected infection ratios $\rho =(\rho_{1}+\rho_{2})/2$ for multiplex networks as a function of parameters *β*_1_ and *β*_2_. The white lines in the lower left corner are used to separate the epidemic survival region from epidemic die out region. *ρ* = 0 in this lower left smaller region means that the epidemic is eventually dying out. ρ>0 in the upper right region means that the epidemic is going to be eventually persistent in the population. Thus, horizontal and vertical white lines represent the epidemic thresholds of complex network *A* and complex network *B*, respectively. Comparing the horizontal and vertical white lines in Figs. 5–7, we show that epidemic thresholds are decreased for cooperative multiplex networks using both first order approximation and second order approximation as compared to the epidemic thresholds of corresponding isolated networks. At the same time, Figs. 5 and 6 show that the die out regions differ trivially between first and second order approximation. Therefore, the simulation results are consistent with that of the previous simulations.

Finally, we use simulations for sensitivity analysis of parameters. The second order approximation for cooperative spreading processes is used in numerical simulations. Considering the interacting network *A*, the comparison of *ρ*_1_ for different values of *μ*_1_ with $\gamma_{1}=0.04$ is shown in Fig. 8, which reveals that larger *μ*_1_ leads to a smaller final infection fraction. Further, the comparison of *ρ*_1_ for different values of *γ*_1_ with $\mu_{1}=0.4$ is shown in Fig. 9. Simultaneously, we can also obtain similar results which were not displayed for interacting network *B*. The same observation for different parameters is obtained, which reveals that the conclusion is not sensitive to parameters.

## IV. CONCLUSION

In conclusion, a model for cooperative spreading processes on interacting two-layer networks is proposed. In particular, the epidemic thresholds of interacting two-layer networks can be decreased for cooperative spreading processes, thereby implying that cooperative spreading processes promote the spread of the disease. In theory, the global epidemic onset arises in the interacting networks simultaneously. Thus, a global epidemic threshold can be used to uniform the two epidemic thresholds. This model can also be used for unidirectional coupled networks. The results can provide hints for public health authorities to make effective measures for disease control and prevention.
